# Exploiting Autophagy-Dependent Neoantigen Presentation in Tumor Microenvironment

**DOI:** 10.3390/genes14020474

**Published:** 2023-02-13

**Authors:** Evangelos Koustas, Eleni-Myrto Trifylli, Panagiotis Sarantis, Nikolaos Papadopoulos, Konstantinos Papanikolopoulos, Georgios Aloizos, Christos Damaskos, Nikolaos Garmpis, Anna Garmpi, Dimitris Matthaios, Michalis V. Karamouzis

**Affiliations:** 1 Department of Biological Chemistry, Medical School, National and Kapodistrian University of Athens, 11527 Athens, Greece; 2 First Department of Internal Medicine, 417 Army Equity Fund Hospital, 11521 Athens, Greece; 3 ‘N.S. Christeas’ Laboratory of Experimental Surgery and Surgical Research, Medical School, National and Kapodistrian University of Athens, 11527 Athens, Greece; 4 Renal Transplantation Unit, ‘Laiko’ General Hospital, 11527 Athens, Greece; 5 Second Department of Propaedeutic Surgery, ‘Laiko’ General Hospital, Medical School, National and Kapodistrian University of Athens, 11527 Athens, Greece; 6 First Department of Pathology, Medical School, National and Kapodistrian University of Athens, 11527 Athens, Greece; 7 Oncology Department, General Hospital of Rhodes, 85100 Rhodes, Greece

**Keywords:** autophagy, cancer, immunotherapy, neo-antigen, tumor microenvironment

## Abstract

Autophagy constitutes a well-known homeostatic and catabolic process that is responsible for degradation and recycling of cellular components. It is a key regulatory mechanism for several cellular functions, whereas its dysregulation is associated with tumorigenesis, tumor–stroma interactions and resistance to cancer therapy. A growing body of evidence has proven that autophagy affects the tumor microenvironment, while it is also considered a key factor for function of several immune cells, such as APCs, T-cells, and macrophages. Moreover, it is implicated in presentation of neo-antigens of tumor cells in both MHC-I and MHC-II in dendritic cells (DCs) in functional activity of immune cells by creating T-cell memory, as well as in cross-presentation of neo-antigens for MHC-I presentation and the internalization process. Currently, autophagy has a crucial role in immunotherapy. Emergence of cancer immunotherapy has already shown some remarkable results, having changed therapeutic strategy in clinical practice for several cancer types. Despite these promising long-term responses, several patients seem to lack the ability to respond to immune checkpoint inhibitors. Thus, autophagy through neo-antigen presentation is a potential target in order to strengthen or attenuate the effects of immunotherapy against different types of cancer. This review will shed light on the recent advances and future directions of autophagy-dependent neo-antigen presentation and consequently its role in immunotherapy for malignant tumors.

## 1. Introduction

Autophagy is a complex primary homeostatic pathway based on a lysosomal-degradation mechanism that reassures favorable conditions for cells under stressful conditions, such as lack of nutrients and oxygen, as well as in case of aggregation of several misfolded proteins and defected organelles thatare subject to degradation and recycling. This mechanism is considered pivotal for maintenance of several cellular functions, whereas its deregulation potentially leads to carcinogenesis and resistance to anti-neoplastic therapeutic modalities, as well as being associated with interactions between atumor and its stroma. In addition, autophagy closely regulates the anti-tumor immune response and tumor microenvironment, while it must be underlined that the autophagy mechanism stimulates immune responses to a great degree when it antecedes apoptosis [1].

Tumor microenvironment (TME) constitutes a complex entity that supports and surrounds tumor cells, which is composed of an extracellular matrix and several types of cells, including stromal and immune cells, as well as blood vessels [2]. There is an emerging role between TME and immune responses as it contains several potentially druggable targets, while its components have a key role in tumor progression. TME is characterized by a great extent of cellular heterogeneity as it is comprised of several types of cells, includingcancer-associated fibroblasts (CAFs), myeloid-derived suppressor cells (MDSCs), tumor-associated macrophages (TAMs), as well as tumor-infiltrating lymphocytes (TILs) and T/B regulatory (Breg/Treg) cells. Additionally, it contains several molecules that are secreted by tumor cells, such as cytokines and various growth factors [2,3].

Anti-cancer immune response is a complex procedure that is comprised of three discrete steps: the asymptomatic phase, in which the innate and adaptive immune systemsidentify tumor cells and reassure their elimination via the cytotoxic effect of immune cells and antibodies production against neoantigens, respectively. Subsequently, there is the second phase of balance, in which tumor cells escape immunosurveillance, making their limitation impossible. This leads to the third phase of the tumor escape mechanism, which is characterized by further tumor growth and progression. Based on the aforementioned, identification and limitation of tumor neoantigensare considered potent weapons against the tumor escape phenomenon and are in the research spotlight [3].

Recent studies have demonstrated the interplay between anti-tumor immune responses and autophagy via several mechanisms, including degradation of NK-cells-derived granzyme B, which otherwise induces a cytotoxic effect on tumor cells, as well as enhancement of inhibitory checkpoint inhibitors’ (ICIs) expression, such as cytotoxic T-lymphocyte-associated protein-4 (CTLA-4) and programmed cell death protein (PD-1) via autophagy in malignant cells. Autophagy acts in dendritic cells as a tumor suppressor via increasing antigen presentation and T-cells’ cytotoxic effects, leading to tumor growth limitation [4].

A better understanding of autophagy machinery and tumor escape mechanisms, as well as a complete understanding regarding tumor microenvironment (TME) implications on efficacy of anti-neoplastic therapies, reveal new horizons for more efficient management of cancer patients.

## 2. A Synopsis of the Macroautophagy Mechanism

Autophagy constitutes a multiphasic catabolic process that reassures cell homeostasis via a lysosomal degradative system. As previously indicated, cell survival is closely associated with autophagy under stressful conditions, such as accumulation of defective organelles and proteins or during oxygen and nutritional deprivation. There is a sequence of highly regulated steps that lead to autophagosome formation and maturation and finally its degradation [5]. Elucidating this multi-stepped mechanism, there are five distinct steps, including (i) the induction step by which the autophagy pathway is initiated via inhibition of the mammalian target ofrapamycin (mTOR), followed by activation of Unc-51-like kinase 1 complex (ULK1). This complex is comprised of FIP200, ATG13, ATG101, and ULK1, which have a key role in phosphorylation (activation) of class III PI3K. In the first step, the phagophore is empty, while, afterward, cargo starts to be engulfed. The next step is (ii) phagophore nucleation, in which class III PI3K is activated via ULK1 and then forms a complex with Beclin-1. Afterward is (iii) elongation, in which the phagophore is elongated, resulting in formation of an autophagosome. For maturation of the latter, two conjugations are required: the first between ATG12 and ATG5 and the second between microtubule-associated protein 1 light chain 3 I (LC3I) and lipid Phosphatidylethanolamine (PE).The former requires activation of ATG12 by ATG7, which also activates LC3, followed by formation of thioester intermediates between ATG12 and ATG10. Later, ATG12 is conjugated with ATG5, resulting in formation of the ATG12–ATG5 complex. Additionally, LC3 is cleaved in order to form LC3I and subsequently activated by ATG4 and ATG7, respectively. Conjugation of LC3I with PE requires implication of ATG12–ATG5 complex and ATG3, resulting in formation of the lipidated LC3 form, the LC3II [6].

**Figure 1:**
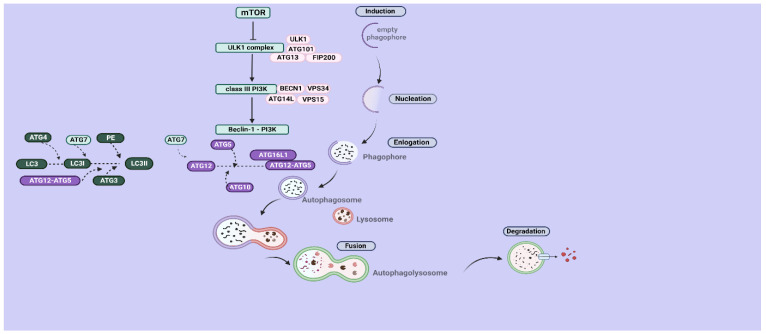
A schematic presentation of the macroautophagy pathway.Autophagy includes five major steps: (i) induction, (ii) nucleation, (iii) elongation, (iv) fusion, and (v) degradation. After mTOR is deactivated and ULK1 activated, cargo starts to become engulfed inside the previously empty phagophore and later nucleated, which requires activation of classIII PI3K by the ULK1 complex. Later, the phagophore is elongated, which is mediated via formation of the classIII PI3K- Beclin1 complex, resulting in formation of the autophagosome. Subsequently, the autophagosome is matured by two conjugation reactions and later fused with lysosomes, which leads to its degradation [3,4,5,6,7]. This figure was created with BioRender.com (agreement number PU24S95UJE).

Furthermore, the aforementioned conjugations lead to autophagophorematuration and then the (iv) fusion step follows, by which lysosomes are united with autophagosome, forming autophagolysosome, which is subject tothe last step:(v) degradation [7]. We present a schematic presentation of the macroautophagy pathway in Figure 1.

## 3. The Role of TME Components

TME is a dynamic system surrounding amalignant tumor thatis comprised of a wide variety of tumor-secreting molecules and cells, while it has a pivotal role in resistance to therapeutic modalities, intravasation, and extravasation of tumor cells, as well as in neoangiogenesis and metastatic dissemination of malignant cells [8]. In this section, we will shed light on the role of each TME component and its implication in tumor progression.

The pivotal components of TME are immune cells, which can significantly enhance tumor progression or suppress it, such as T-cells, macrophages, natural killer (NK) cells, neutrophils, and dendritic cells. These cells constitute the innate immune cells in TME, whereas B-cells are adaptive immune cells that produce several antibodies against the neoantigens on the surface of tumor cells. Neoantigens are recognized by T cytotoxic cells (CD8-positive) that are included in TME, a phenomenon that is mediated by the T-cell receptor (TCR) that interacts with MHC-I tumor-associated antigens. Presence of CD8-positive T-cells has been related to relatively better prognosis via its major role in tumor cell lysis and release of interferon-γ (IFN-γ), withthe latter restricting neoangiogenesis [9,10].

Moreover, NK cells also induce tumor cell destruction directly or indirectly via release of several cytokines in the bloodstream, resulting in restriction of tumor progression [11]. Meanwhile, neutrophils have a binary role, eithersuppressing tumor progression in the early stages or promoting tumor growth via release of several molecules, such as matrix metalloprotease (MMP) −9 and vascular endothelial growth factor (VEGF), which further promote alterationinextracellular matrix consistency, invasion of tumor cells in the neighbor tissues, and neoangiogenesis [12]. In addition, there are two subpopulations of neutrophils: (i) N2 that promotes tumor progression via release of the aforementioned molecules, and (ii) N1 that restricts immunosuppression via release of intercellular adhesion molecule 1(ICAM-1), tumor necrosis factor-α(TNF-a), as well as production of reactive oxygen species(ROS) [13].

Other subdivisions of the T-cell population are conventional helper cells (CD4-positive), which regulate TH-2 activation, as well as T regulatory cells (Tregs). The former subpopulation releases several proinflammatory cytokines, while the latter constitutes a key player for tumor progression via suppressing anti-cancer immunity. Tregs are originated either by T-cells differentiation (peripherally) or by the thymus, while they are characterized by overregulated expression of different molecules, such as FOXP3, TGF-β, IL10, IL-2, IL-35, as well as PD-1 and CTLA-4 immune checkpoints [14,15,16]. This phenomenon is closely associated with interaction between Tregs and tumor cells, with release of IL-2 that deregulates NK cell function, as well as with suppression of CD8-positive T-cells via FOXP3 expression. Moreover, Tregs closely interact with other TME components, such as the fibroblast, endothelial cells, and stromal cells, a phenomenon that induces development and growth of tumor cells.

Meanwhile, B-cells have a key role in antibody production against neoantigens in order to induce tumor cell elimination; however, Bregs are closely associated with tumor progression via their inhibitory effect on innate immune cells [17]. TAMs constitute some other immune modulatory cells that are closely related to worrisome prognosis via enhancement of neoangiogenesis and cytokine production [18]. More particularly there are two distinct subcategories of TAMs: (i) M1-anti-tumorigenic and (ii) M2-protumorigenic, while TAMS together with myeloid-derived suppressor cells (MDSCs) notably enhance tumor growth and progression via promoting epithelial–mesenchymal transition and neoangiogenesis [19].

Additionally, MDSCs are also considered tumor promoters via their immunosuppressive effect, as well as through their tumor-mediated regulation via CCL5 and CCL2 tumor-secreted molecules [20], whereas DCs constitute antigen-presenting cells (APCs), which facilitate antigen identification by CD8-positive T-cells [21].

Furthermore, CAFs are included in tumor stroma, inducing their production via conversion of tissue-fibroblasts under the influence of several growth factors, resulting in ECM modifications. In addition, they also have a binary role in tumor progression, either acting as tumor suppressors via secretion of transforming growth factor β (TGF-β) that leads in metastasis restriction [22] or as promoters oftumor growth, invasion, and dissemination. These phenomena are attributed to ECM modification by metalloproteinase MMP-3 that degrades E-cadherin, while neoangiogenesis is mediated through VEGF release by ECM.

Additionally, ECM composition is closely related to desmoplasia, which mediates anti-cancer therapy resistance. Some of the ECM components are collagen and elastin fibers, together with CAFs, fibroblast growth factors (FGF), TGF-β,VEGF, and platelet-derived growth factor (PDGF), which constitute tumor-promoting substances and promote desmoplasia [23].

Another TME cell entity is endothelial cells (ECs), which reassure blood supply for agrowing tumor under the stimulatory effect of VEGF, promoting neoangiogenesis [24]. The new tumor vasculature does not have the proper structure andintercellular connections (“leaky vessels”), a condition that promotes extravasation of tumor cells and formation of distant metastasis [25]. However, ECs are also implicated in endothelial–mesenchymal transition (EMT) phenomenaand are further transformed into CAFs [26].

**Figure 2:**
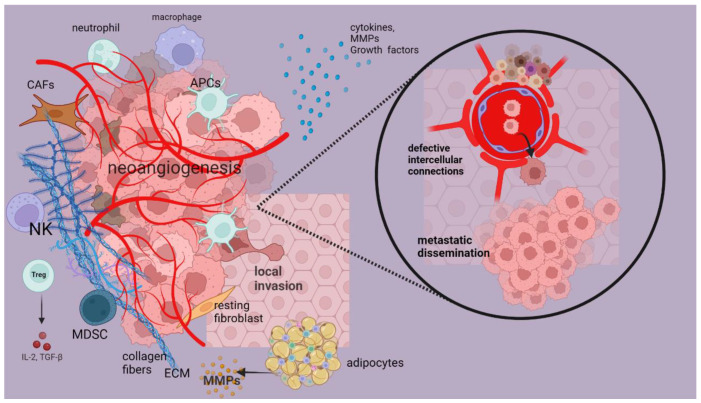
A schematic presentation of TME implications in tumor growth and progression.ECM is characterized by desmoplasia, which results from aggregation of collagen/elastin fibers and CAFs. Endothelial cells (ECs) are related to neoangiogenesis under the stimulatory effect of VEGF. The new vasculature lacks proper intercellular connections (“leaky vessels”), permitting extravasation of tumor cells and formation of distant metastasis. Adipocytes induce ECM alteration via release of MMPs, while neutrophils also secrete MMP-9 and VEGF, inducing ECM modification, neoangiogenesis, and local invasion. NK cells secrete cytokines or directly induce tumor cell destruction, while Tregs release several cytokines, such as IL-2, which deregulates NK cells, while TAMs secrete cytokines that enhance angiogenesis. CAFs are produced via conversion of tissue-fibroblasts and degrade E-cadherin in ECM via MMP-3, promoting tumor invasion [22,23,24,25,26,27]. This figure was created with BioRender.com (agreement number AD24S95KVO).

Finally, adipocytes areincluded in tumor stroma and have major roles in tumor progression via releasing MMP-14,7,10, 1, and 11 that alter the ECM [27]. In Figure 2, we present a schematic presentation of TME components and their implication in tumor progression.

## 4. An Overview of Anti-Tumor Immunity

Anti-tumor immunity constitutes an adaptive immune response, which is stimulated by several tumor-associated antigens (TAAs) or tumor-specific antigens (TSAs), aiming at tumor limitation and destruction [28]. The latter category of tumor antigens, such as cancer-germline genes, is only found in malignant cells and mostly associated with oncogenic mutations and oncoviruses [29]. Meanwhile, the former antigens are overexpressed in malignant tissue; however, they are also found to alesser extent in normal state [30].

The anti-tumor immune response is a complex procedure, which includes T cytotoxic cells immunosurveillance, neoantigen presentation by APCs, and aninteraction between T lymphocytes and tumor antigens, as well as activation of T effector cells, including Th1 and Th2 cells, that activate macrophages and B-cells, respectively. More specifically, the interaction between T cytotoxic (CD8+)cells and tumor cells is mediated via T-cell receptor (TCR) and MHC-type I molecules, which presents tumor antigens that are intracellularly originated. On the other hand, presentation of extracellular antigens is mediated via MHC-type II, which interacts with the TCR of CD4+ T-cells. However, it must be underlined that, under circumstances of modified MHC expression, the antigen presentation is compromised, leading to tumor development and immunoresistance [31].

## 5. Autophagy-Dependent TME Modulation and Neoantigen Presentation

### 5.1. Autophagy-Dependent Neoantigen Presentation

There are several studies that illustrate the influence of the autophagy pathway in TME. This pathway has a significant impact on anti-tumor immune response and also alters tumor stroma [32]. When there are stressful conditions in the TME, the autophagy pathway is initiated in order to maintain cell survival. Induction of the pathway can either promote or suppress tumor progression, while it also has a dual role in antigen presentation, which can either be increased or decreased, leading to enhanced T-cytotoxic cell stimulation that suppresses tumor progression and tumor escape mechanism, respectively [32,33]. More particularly, autophagy enhances the procedure of antigen trafficking to the endosomal network in order to be degraded and later loaded on MHC for itspresentation.

Moreover, autophagosomes are significantly implicated in loading of antigens on MHC I/II, which finally interact with the TCR receptor. Intracellular antigens, in order to be transferred on the APCs surface, must be isolated and degraded under action of lysosomal enzymes that are enclosed inautophagolysosome, producing several peptides, which are further integrated with MHCII [34]. In addition, it must be noted that this procedure requires participation of several other protein molecules, such as calreticulin, ERp60, as well as tapasin [35].

Meanwhile, immunosurveillance of antigens that have exogenous origin requires their transfer to ER by LC3-associated phagocytosis (LAP), which constitutes the non-canonical autophagy pathway, and then their binding on the MHC molecule. The aforementioned mechanism enables recognition of antigens by several surface receptors, such as toll-like receptors (TLRs), TIM4, pattern recognition receptors (PRR), as well as Fc receptors [36].

### 5.2. Autophagy-Dependent TME Modulation

Autophagy is closely interrelated with TME as it is suppressed under an inflammatory state, which promotes dysplasia and tumorigenesis. Under inflammatory conditions, immune cells that are infiltrated in the tumor secrete several cytokines and inflammatory mediators, such as IL-6, (TGF)-β, IL-1b, as well as TNF-A and IL-10, while a significant amount of reactive oxygen species (ROS) areproduced and activated in the TME [37]. The aforementioned phenomenon leads to autophagy induction. However, there are studies that demonstrate that suppression of autophagy-related genes in breast cancer stem cells leads to reduced cytokine secretion and suppressed STAT3 signaling pathway, which leads to limitation of tumor growth and progression [38]. Moreover, tumor cells are frequently under conditions of oxygen and nutritional deprivation, which leads to autophagy activation via PKC-augmented JNK activation, hypoxia-induced factor-1 α (HIF-1α), AMPK activation, and mTOR inactivation, respectively [39].

Furthermore, autophagy significantly alters immune responses because it is closely associated with the functional states of several immune cells, such as MDSCs, DCs, as well as NK cells and T-lymphocytes [40].

Finally, autophagy is also associated with immune checkpoints, which are expressed on the surface of malignant cells, with overexpression of PD-L1 being related to mTOR activation and autophagy downregulation [41].

### 5.3. Autophagy-Dependent Neoantigen Presentation—Tumor Progression

The autophagy mechanism is significantly implicated in established tumors that escape immune surveillance. This lysosomal-degradative homeostatic mechanism is closely associated with the functional state of innate immune cells, such as macrophages, NKs, T-cells, as well as APCs, such as dendritic cells (DCs) [42].

#### 5.3.1. Autophagy Implication in Macrophage-Mediated Immune Surveillance

Macrophage-mediated phagocytosis has an important role in immune surveillance in malignant tumors. However, it is demonstrated that phagocytosis is notably impaired, a phenomenon that is mainly attributed to the autophagy mechanism in cancer cells [43].

#### 5.3.2. Autophagy Implication in NKs Cytotoxic Effect

In the level of NKs’ functional state, autophagy inducesattenuation of the anti-neoplastic effect of these cells via degrading granzyme B, which is enclosed in their granules and has a pivotal lytic effect against tumor cells. However, its degradation promotes tumor cells escape from the cytotoxic effect of NKs.

Meanwhile, HIF-1a stimulates autophagy and promotes tumor survival under oxygen deprivation. This phenomenon leads to autophagy-induced degradation of granzyme B and impairment of NKs function [44,45]. Similarly, impairment of NK tumor cytotoxicity is demonstrated in melanoma through the hypoxia-induced autophagy mechanism, which mediates degradation of wild-type Gap junction α-1 protein (GJA1)(connexin 43 (CX43)), a key substance of intercellular gap junctions [46].

#### 5.3.3. Autophagy Implication in MDSCs Immune Surveillance

Moreover, tumor-associated autophagy is closely related to tumor resistance to immune surveillance, in which TME components, such as Tregs and MDSCs, have a key role in the tumor escape phenomenon, while their survival is closely associated with HMGB1-induced autophagy [47].

Furthermore, autophagy particularly alters MDSCs’ metabolic state and lifespan, as well as their development, while it is crucial for MDSCs regulation. It is demonstrated that autophagy’s impact on MDSCs modulation is of great importance via suppressing anti-neoplastic immunity, whereas autophagy suppression limits tumor growth and enhances immunity against malignant cells [48].

#### 5.3.4. Autophagy Implication in APCs Immune Surveillance and MHC-I/II Regulation

In addition, autophagy can also influence recruitment of APCs cells inside the tumor bed, as well as their maturation. This phenomenon leads to tumor evasion from immune surveillance via autophagy-mediated activation of the STAT3 signaling pathway [49].

Likewise, autophagy constitutes a major regulator of MHC-I/II protein molecules, which significantly facilitate antigen presentation, aiming at T-cell activation. However, these molecules go through autophagy degradation. More particularly, MHCII is degraded by membrane-associated RING-CH1 (March1)E3 ubiquitin ligase in MDSCs, whereas MHCI autophagic degradation is induced by NBRI, with both conditions resulting in tumor immune evasion. Similarly, MHCI degradation in DCs is mediated by adaptor-associated protein kinase 1 (AAK1), which is involved inreceptor-mediated endocytosis (RME). More particularly, AAKI induces endocytosis of MHC1, leading to impaired antigen presentation and T-cell stimulation [50,51].

#### 5.3.5. Autophagy Implication in Immune Checkpoint Inhibitors Regulation

Another key role of autophagy is regulation of immune checkpoint inhibitors programmed cell death protein 1/programmed cell death ligand 1 (PD-1/PD-L1). PD-L1 is expressed in malignant cells, which are bound to PD-1 on the T-cell’s surface. This interaction between T-cells and cancer cells via PD-1 and PD-L1, respectively, leads to tumor escape phenomenon and impaired T-cell cytotoxicity. The reduced PD-L1 autophagic degradation is significantly associated with increased tumor growth and progression, as well as with suppression of anti-tumor immunity and tumor immune escape. The aforementioned situation can be induced by PD-L1 palmitoylation, which is mediated by Palmitoyltransferase ZDHHC3, as demonstrated in animal models [52,53,54].

Similarly, binding of CKLF-like MARVEL transmembrane domain containing 6 (CMTM6) proteinto PD-L1 inhibits PD-L1 autophagic degradation and promotes tumor progression and immune evasion. Thus, PD-L1 expression is maintained by CMTM6, preventing its lysosomal degradation and promoting tumor growth. Finally, Sigma1 has a key role in protein homeostasis of malignant cells, such as in PD-L1 expression, while the interaction between these two molecules leads to suppression of PD-L1 autophagic degradation and tumor immune escape phenomenon [55,56,57].

### 5.4. Autophagy-Dependent Neoantigen Presentation—Tumor Suppression

#### 5.4.1. Autophagy Implication in DC Neoantigen Presentation

Autophagy stimulation in dendritic cells (DCs) mediates enhanced antigen presentation and activation of T cytotoxic (CD8+) cells, such as in the case of Lewis lung carcinoma (LLC) and mammary gland tumors in murine. In the aforementioned case, autophagy stimulation is medicated through utilization of α-tocopheryloxyacetic acid, which elevates LC3II levels, enhancesDCs-mediated antigen presentation b to cytotoxic cells, as well as stimulating multiplication of CD8+ cells [58]. Similarly, conjugation of antigens with nano-activators in DCs also leads to enhancement of antigen presentation and stimulation of TILs. Meanwhile, antigen availability and immune recognition can be further enhanced by newly developed epitopes, which are originated by the autophagy mechanism [59,60].

#### 5.4.2. Autophagy Implication in PD-1/PD-L1 Regulation

Additionally, autophagy-mediated regulation of ICs, including PD-1/PD-L1, has a key role in prevention of the tumor escape phenomenon. The aforementioned phenomenon is attributed to PD-L1 expression on malignant cells, which interacts with PD-1 on T-cells, resulting in T-cell suppression and inhibition of T-cell proliferation. Autophagy-mediated regulation of ICs is illustrated by the effect of Huntingtin interacting protein 1 related (HIP1R) regulating lysosomal degradation of PD-L1 via acting as an autophagy receptor for it [61,62]. Thus, induction of PD-L1 degradation suppresses tumor progression and growth by enhancing the cytotoxic effect of T-cells. Nevertheless, malignant cells could induce inhibition of autophagy-mediated PD-L1 degradation. More particularly, genetic modification (glycosylation) of PD-L1 that is induced by cancer cells via EGFR/B3GNT3 axis suppresses this tumor-suppressive effect of autophagy-mediated PD-L1 degradation, promoting tumor growth and evasion of immune surveillance mechanism in breast cancer models [63].

#### 5.4.3. Autophagy Implication in Neoantigen Uptake–Presentation in APCs

Another example of the beneficial role of autophagy in neoantigen uptake and presentation to APCs is application of synthetic nano-DOX in glioblastoma, by which autophagy is initiated. Thus, induction of the autophagy pathway enhances MHC-I complex expression, APCs stimulation, and neoantigen presentation on glioblastoma cancer cells [64]. Meanwhile, autophagy not only promotes MHCI expression on tumor cells but also enhances MHC-II expression in macrophages [65].

#### 5.4.4. Autophagy Implication in Tumor Immune Interface

Furthermore, it is also demonstrated that autophagy is significantly implicated in the tumor immune interface, while autophagy deletion leads to instant development of tumors in experimental models [66]. A pivotal example is a circumstance of spontaneous tumor initiation after BECN1 autophagy-related gene deletion [67]. However, it is evidenced that autophagy suppresses tumor progression only in early stages and not in already established tumors. An example of this phenomenon is autophagy-mediated inhibition of Treg infiltration in murine with Kras mutant lung malignancy, resulting in suppression of tumor development and progression [68].

#### 5.4.5. Autophagy Implication in T Cytotoxic Cell Activation

In addition, it is reported thatadministration of metformin in mice with mammary gland cancerinduces T-cell autophagy, which further stimulates T cytotoxic (CD8+) cells [69]. In Table 1, we present the implication of autophagy-mediated degradation in anti-neoplastic immune responses.

## 6. The Key Role of Mitophagy in Anti-Tumor Immunity

Mitophagy constitutes a selective autophagic homeostatic pathway of eukaryotic cells to eliminate defective mitochondria, while it is sub-classified into two categories of Ub-independent and (Ub)-dependent pathways. It is demonstrated that mitophagy has a crucial role in innate immunity, which is induced by mitochondrial stress, such as ROS production, infections, defective genetic repair mechanisms, genetic mutations, as well as nutritional and oxygen deprivation [70]. It must be underlined that mitophagy also has a binary role in tumor initiation and progression, acting either as a tumor suppressor or tumor enhancer based on the stage and type of the malignancy [71]. More particularly, mitophagy limits ROS in the early stages of tumor initiation, whereas mitophagy in established tumors leads to tumor growth and progression, as well as drug resistance [72].Efficacy of anti-neoplastic chemotherapy and drug resistance are closely associated with autophagy, while mitophagy is utilized by cancer cells in order to settle their drug resistance. More particularly, ATG12 is significantly implicated in the homeostatic mechanism of mitochondria, as well as cell apoptosis [73].

Additionally, it has a significant role in anti-tumor immune responses via mitophagy induction. More particularly, elimination of defective mitochondria and tumor suppression are mediated via the PINK1/PARK2 pathway, in which PRKN/PARK2 is transformed into E3 ligase that ubiquitinates the impaired mitochondrial proteins [74,75].

Impairment inthe aforementioned pathway leads to carcinogenesis, such as pancreatic. Depletion of PINK1 induces the Warburg effect with HIF-1a transcription factor stabilization in glioblastoma cells. In general, loss of PINK1/Parkin expression is closely associated with increased ROS, reduced mitophagy, and HIF-1a stabilization, which induces glycolysis [76].

Meanwhile, induction of the mitophagy pathway in tumor cells that lack STAT3 leads to enhanced antigen presentation for APCs and activation of T-cell [77]. Several genetic mutations that are associated with impaired mitophagy have been reported in several malignancies, such as inactivation/heterozygous deletion of PARK2 gene in intestinal malignancy. Hepatic malignancy is also prevented by FUNDC1-mediated mitophagy, while FUNDC1 deletion promotes tumorigenicity [78].

Furthermore, HIF-1a transcription factor stabilization is the main characteristic of cancer cells under normal oxygen levels, which promotes tumor development and progressionin an oxygen-independent manner. Alterations in expression of BNIP3 and NIX mitophagy receptors induce stabilization of HIF-1a, a phenomenon that induces the so-called Warburg effect.An example of this phenomenon is depletion of the BNIP3 receptor in mice, which induces an increase in ROS mitochondrial levels, limiting mitophagy pathways and promoting breast cancer development and progression [79,80]. Meanwhile, in human mammaryMCF-7 cancer cells, which are characterized by IGF-1 receptor kinase inhibitor resistance, there are lowered BNIP3 expression levels having the same results as above [81].

Finally, removal of the altered mitochondria is mediated via BNIP3-dependent mitophagy and p53-dependent mitophagy, which promote oxygen utilization and limitation of glycolysis [82].

## 7. Future Therapeutic Perspectives Based on Autophagy-Related Neoantigen Presentation

Autophagy is closely associated with the metabolic and functional state of dendritic cells (DC), which can be exploited for development of novel therapeutic strategies that can overcome the immunosuppressive effect of TME on DCs and enhance efficacy of anti-neoplastic therapeutic modalities. More particularly, a novel strategy is utilization of nanomaterials (NMs) that regulate autophagy of DCs and can subsequentlyoptimize anti-tumor immunity. Autophagy is implicated in DCs activation and maturation, as well as in antigen presentationand cytokine release, resulting intumor suppression [83].

Moreover, suppression of autophagy can overpass the phenomenon of autophagy-mediated CX43 degradation, which leads to impaired NK-mediated toxicity of melanoma cells under hypoxic conditions [46]. Additionally, tumor suppression can be achieved via reduction of Beclin1, resulting in intensification of chemokine (C-C motif) ligand 5 (CCL5) expression in melanoma cells, promoting NK-mediated killing of tumor cells [84]. More particularly, tumor growth is inhibited via suppression of macroautophagy/depletion of Beclin1 in melanoma tumors, which induces NK cell infiltration in the TME and release of CCL5, which is strongly correlated with a better survival rate for melanoma patients [85].

As previously mentioned, autophagy constitutes a major regulator of MCH I and II levels, while it induces their degradation, leading to the tumor escape phenomenon. However, depletion of ATG5 can increase levels of MHC-II and subsequently enhance T-cell activation. It is demonstrated that MDSCs that lack autophagy exhibit intensified expression of MHC class II that promotes tumor-specific T helper cells (CD4+), a phenomenon that implies the immunosuppressive effect of autophagy in TME. Additionally, targeting March 1 E3 ligase, autophagic degradation of MHCII is modified, which constitutes a strategy that significantly limits tumor growth and progression, as well as enhancing anti-neoplastic immunity [86].

Furthermore, PD-L1 palmitoylationbypalmitoyltransferase ZDHHC3 (DHHC3), which suppresses its autophagic degradation, can be overcome via inhibiting DHHC3 enzyme (silencing) or via blocking the reaction of palmitoylation by 2-Bromopalmitate. Both strategies lead to increased PD-L1 autophagic degradation and subsequently promote T-cell cytotoxicity against malignant cells [53]. Additionally, another treatment strategy for promotion of PD-L1 autophagic degradation is via utilization of aloperine derivative, the so-called SA-49, an agent that induces the PD-L1 autophagic degradation in a MITF-dependent manner [87].

Similarly, another agent that promotes PD-L1 autophagy lysosomal degradation is multi-targeted receptor tyrosine kinase (RTK) inhibitor sunitinib. Utilization of sunitinib has a noteworthy beneficial impact on tumor immune surveillance via regulating PD-L1 [88]. Regulation of PD-L1 is mediated by the P62 molecule, which is bound to PD-L1 in order to facilitate its transfer in the site of lysosomal degradation [89].

Further, the aforementioned strategies for promotion of PD-L1 autophagy degradation, CMTM6, and PD-L1 binding can be inhibited either via H1A anti-PD-L1 or via CMTM6 reduction that notably reduces impairment inT-cell cytotoxicity [90]. Meanwhile, inhibition of SIGMA1, which interacts with PD-L1, potentially leads to activation of T-cells [57]. Meanwhile, degradation of PD-L1 and stimulation of T-cell activity arealso mediated by verteporfin [91].

Finally, mitophagy is a homeostatic mechanism degradation pathway for defective mitochondria. There are several common signals that trigger not only mitophagy but also carcinogenesis and cellular death. This phenomenon provides several therapeutic targets for selective destruction of malignant cells. Mitophagy constitutes an extra weapon for eradication of cancer cells via utilization of mitophagy activators or suppressors. An example of mitophagy inducers is deferiprone and phenanthroline, with the latter being a sodium-channel inhibitor and the former constituting an iron chelator. Moreover, induction of mitophagy is mediated via hydrolysis of linamarin to cyanide, glucose, and acetone by enzymatic action of linamarase. Then, cyanide suppresses cytochrome c oxidase, resulting in activation of the mitophagy pathway, while the pivotal energy-making pathways, such as oxidative phosphorylation and glycolysis, are suppressed [92].

Meanwhile, it is demonstrated that endogenous or exogenous stimulation of mitophagy by ceramides induces cancer cell death via the autophagy pathway. Similar effects are reported for sphingolipids that also induce mitophagy-associated cell death [93,94].

Furthermore, there are many mitophagy suppressors, such as liensinin, cyclosporine-A, and mitochondrial division inhibitor-1, which inhibit the double-sword action of mitophagy by blocking mitochondrial degradation [92].

It has to be underlined that cancer cells, in order to progress and develop drug resistance, use the mitophagy pathway to eliminate defective mitochondria. Drug resistance to several chemotherapeutic agents, such as paclitaxel, cisplatin, 5-fluorouracil, and doxorubicin, is often demonstrated in mitophagy, which either promotes or suppresses survival of cancer cells [95].

Finally, autophagy can be utilized as a facilitator of chimeric antigen receptor T (CAR T) cell-associated cytotoxicity in hematological malignancies of B-cells. More particularly, despite the promising anti-neoplastic effect of CAR-T cells in hematologic malignancies, utilization of this modality is still limited due to the resistance of cancer cells and the subsequent failure of the treatment. However, this phenomenon can be overcome by adding an autophagy inhibitor, such as autophinib in malignant B-cells culture, as observed in vitro. It is reported that autophagy suppressor induced the cytotoxic effect of the CAR T-cells against CD19 by inducing degradation of caspase 9 and 8. However, further research is needed on the combination of autophagy modulators in CAR-T cell treatments [96]. In Table 2, we present the implication of autophagy/mitophagy and crosstalk with anti-neoplastic immunity in several malignancies.

## 8. Conclusions

Autophagy constitutes a pivotal homeostatic process that permits stability for several cellular components of tumor milieu, which further leads to immunosuppression in TME. The immunosuppressive character of TME is mediated by autophagy, which influences recognition of neoantigens, as well as their presentation to APCs, their recognition, as well as immune cell recruitment. Targeting autophagy can potentially overcome immune-resistant TME, while it is considered efficacious as it enhances immunotherapy efficacy. However, it was underlined that autophagy has a binary role in tumor immunogenicity, which can lead either to tumor growth or tumor suppression. Further research is considered pivotal for identification of specific autophagy-related genes that are closely associated with tumor immune surveillance, while it is considered critical for development of novel therapeutic strategies, including autophagy inhibitors or promoters, personalized for each tumor based on the stage and phenotype of TME.

## Figures and Tables

**Table 1:** Autophagy-mediated degradation and anti-neoplastic immune responses [50,51,52,53,54,59,60,61,62,63,64,65].

Autophagy-Mediated Degradation	Results
Degradation of MHC-I	Impaired antigen presentation
by NBR1	Impaired T-cell activation
	Tumor immune evasion
	
Degradation of MHC-I in DCs	Impaired antigen presentation
by AAK1	Impaired T-cell activation
	Tumor immune evasion
	
Degradation of MHC-II in MDSCs	Tumor immune evasion
by March1 E3 ubiquitin ligase	
	
Degradation of PD-L1	
HIP1R (autophagy receptor) binds PD-L1	Suppression of tumor growth and progression
	Promoted T-cell cytotoxicity
	
	
Impaired degradation of PD-L1	Impaired T-cell cytotoxicity
via CMTM6–PD-L1 binding	Tumor immune evasion
via PD-L1 palmitoylation	
via SIGMA1-PD-L1 interaction	
	

**Table 2:** Implications of autophagy/mitophagy and crosstalk with anti-neoplastic immunity in several malignancies [43,97,98].

Malignancy	Autophagy Regulation	Role of Autophagy in Anti-Tumor Immunity	Mechanism
Gastric cancer cells	↑	Activated	↓ expression of PD-L1
Glioblastoma cells	↑	Activated	↑ Immunogenic cell death
Pancreatic cancer cells	↑	Inhibited	↑ degradation of MHC-type I
Melanoma	↓	Inhibited	↓ T cytotoxic cells proliferation
			↑ secreted INF-γ
			↓ killing effect of T cytotoxic cells
		
	↑	Inhibited	↓ infiltration/killing effect of NK cells
			↑ suppressive effect of MDSCs
		
Endometrial cancer cells	↑	inhibited	↓ expression of MHC
Intrahepatic cholangiocarcinoma	↑	Activated	↑ PD-L1 degradation
Breast cancer (triple negative)	↓	Inhibited	↓ killing effect of T cytotoxic cells
	↑	Activated	↑degradation of PD-L1
			
Breast cancer			
	↑	Inhibited	↑ suppressive effect of MDSCs
			↓ killing effect of NK cells
Hepatocellular carcinoma	↑mitophagy	Activated	Overregulation of Dendritic and cytotoxic T-cells
Renal Cell Carcinoma, Colorectal	↑	Inhibited	TANs activation, tumor migration ↑ and metastatic dissemination ↑
melanoma			

Colorectal cancer	↑	Activated	
			↑ apoptosis of TAMs, ↓ tumor proliferation, ↑ radiosensitivity of CRC

			↓ IDO
			production,
			enhancement of anti-tumor immunity



Lung cancer			↑ Treg infiltration, immune suppression
Non-small-cell lung cancer			↓ T-cells activation- Killing effect of T cytotoxic cells
	↑	Inhibited	

↑: upregulation; ↓: downregulation.

## Data Availability

Not applicable.
